# Epileptic seizure, as the first symptom of hypoparathyroidism in children, does not require antiepileptic drugs

**DOI:** 10.1007/s00381-016-3264-2

**Published:** 2016-12-12

**Authors:** Meng-Jia Liu, Jiu-Wei Li, Xiu-Yu Shi, Lin-Yan Hu, Li-Ping Zou

**Affiliations:** 1 Department of Pediatrics, Chinese PLA General Hospital, 28 Fuxing Road, Beijing, 100853 China; 2 Department of Neurology, Beijing Children’s Hospital, Beijing, 100045 China; 3 Center of Epilepsy, Beijing Institute for Brain Disorders, Beijing, 100000 China

**Keywords:** Epilepsy, Parathyroid, Intracranial calcification, Hypocalcemia

## Abstract

**Objective:**

Patients with hypoparathyroidism exhibit metabolic disorders (hypocalcemia) and brain structural abnormalities (brain calcifications). Currently, studies have determined whether antiepileptic drug (AED) treatment is required for epileptic seizures in children with hypoparathyroidism.

**Method:**

This study aims to evaluate the data of two medical centers in Beijing based on the diagnosis of epileptic seizures as the first symptom of hypoparathyroidism in children.

**Result:**

A total of 42 patients were included and assigned into AED and non-AED treatment groups in a 1:2 matched case–control study. Results show that the seizure outcome after 1 year of AED treatment is not significantly different from that of the control. In the subgroup analysis of patients with subcortical calcifications, the seizure outcome is still not significantly different from that of the control.

**Conclusion:**

Thus, AED treatment cannot improve the seizure outcomes in children with parathyroid disorder, even in such cases as suspected structural seizure caused by subcortical calcifications. Clinicians must take adequate considerations on the use of AEDs in these patients. Epileptic seizures, as the first symptom of hypoparathyroidism in children, do not require epilepsy drugs.

## Introduction

Epileptic seizure occurs when a burst of electrical impulses in the brain exceeds the normal limits. Its manifestation can vary from uncontrolled jerking movement (tonic–clonic seizure) to momentary loss of awareness (absence seizure). These impulses spread to adjacent areas in the brain and create an uncontrolled storm of electrical activity. Brain diseases characterized by enduring predisposition to generate epileptic seizures are collectively called epilepsy. According to pathogenesis, epilepsy can be classified into six categories: metabolic, structural, inherited, immunologic, inflammatory, and idiopathic.

Hypoparathyroidism is an endocrine disease that results from parathyroid hormone (PTH) deficiency or any resistance to its hormonal function, and manifestations of this disease mainly include hypocalcemia, hyperphosphatemia, and abnormal PTH serum level [1]. Seizures in patients diagnosed with hypoparathyroidism are ascribed to a calcium phosphorus metabolism disorder. In addition, parathyroid disorders are usually accompanied by intracranial calcifications, which occur in 21.5 to 73 % of patients with hypoparathyroidism; these observations are recognized as long-term complications of hypocalcemia [2]. Hypocalcemia can lead to reduced excitatory threshold, increased neural transmission, and increased neuromuscular excitation [3]. This condition can also lead to the increased susceptibility of hippocampal neurons to epilepsy and can impair the cerebral function through encephaledema, increased intracranial pressure, and metabolic disorder [4]. In this case, a sole treatment with antiepileptic drugs (AEDs) is insufficient. Combining calcium supplements and AEDs is necessary to produce the optimal outcome in patients with epilepsy. Meanwhile, structural etiology refers to lesions in the brain, including intracranial calcifications. The probability of intracranial calcification occurrence is approximately 0.36/12,000. In the studies on Sturge–Weber syndrome, intracranial calcification is thought to be closely related to the severity of epilepsy [5]. Other studies demonstrated that the location of subcortical calcification corresponds to that of the epileptic discharge and that the severity of epilepsy is related to gliosis around the calcification [6, 7].

For epilepsy caused by intracranial calcification, the use of AEDs remains controversial among clinicians. Moreover, few studies with large sample size have been conducted to address this problem, and most of the related studies available now are case reports. We retrospectively reviewed the information from two medical centers in Beijing to address this problem and to provide guidance to clinicians.

## Method

We retrospectively reviewed 42 patients diagnosed with parathyroid disorders who exhibited epileptic seizure as a first symptom. These patients were introduced in the pediatric departments of PLA General Hospital and Beijing Children’s Hospital from 2001 to 2015. Considering that the main outcome is the prognosis after treatment with AEDs for 1 year and both groups (AED group and non-AED group) received calcium supplement therapy, we conducted a 1:2 matched case–control study by length of the course and calcium supplement therapy. The ethics committees of the participating institutions approved this study.

**Fig. 1:**
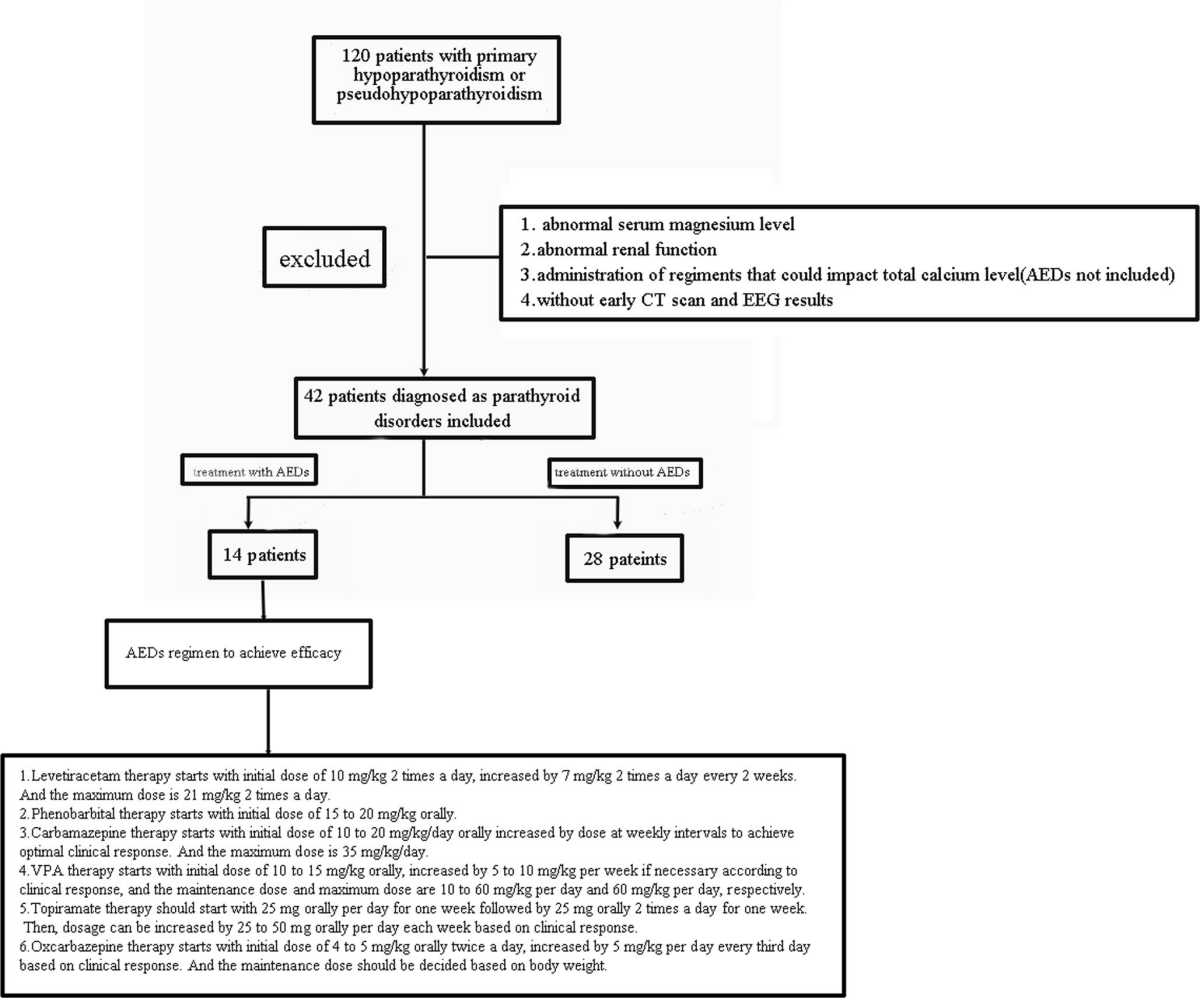
Participant flow

The diagnosis of parathyroid disorders was conducted through blood and urine calcium phosphorus metabolism and PTH serum level tests. Other inclusion criteria were as follows: all the patients underwent CT scan and EEG test, and the results showed existence of intracranial calcifications, the first manifestation was recurrent seizures, and all the patients had received calcium supplement therapy, and the dosage adjustment was made through blood calcium level monitoring. Patient flow and AED regimen are shown in Fig. 1.

The variables that we collected include sex, results of CT scan, EEG results, use of AEDs, type of parathyroid disorders, Chvostek’s sign, Trousseau’s sign, and calcium phosphorus metabolism test results.

### Statistical analysis

All analyses were performed with SPSS 19.0. The paired-sample *t* test was used for the comparison of the continuous variables. For categorical variables, univariate conditional logistic regression was used to determine the odds ratio (OR). A probability value of *P* < 0.05 was considered statistically significant. The ORs and 95 % confidence intervals (95 % CIs) were presented.

## Results

### Patient characteristics

As shown in Fig. 1, 42 of the 120 patients with parathyroid disorders satisfied the criteria. They were divided into the AED treatment and non-AED treatment groups in a 1:2 matched case–control approach. The characteristics of the 42 patients are shown in Table 1. Generally, 22 (52.38 %) of the patients are males, and 20 (47.62 %) are females. The mean age at the onset is 7.55 (1 month to 16 years old). Furthermore, 25 (59.52 %) and 17 (40.48 %) patients were diagnosed with hypoparathyroidism and pseudohypoparathyroidism, respectively. The seizures lasted from several seconds to around 30 min, as shown in Table 2, and most of the seizures exhibited convulsions (hypoparathyroidism 72 %, pseudo-hypoparathyroidism 58.8 %). Relatively few tetany episodes were observed (hypoparathyroidism, 20 %; pseudohypoparathyroidism, 11.8 %). However, numbness, dizziness, and absence were rare. Incidence of mental retardation was observed in 24 and 11.8 % of the patients with hypoparathyroidism and pseudohypoparathyriodism, respectively. Patients with Albright’s hereditary osteodystrophy exhibit a rounded face, short stature, mild mental retardation, and brachydactylia. These cases composed 23.5 % of the patients with pseudo-hypoparathyroidism. Incidence of QT interval prolongation was 40 % in hypoparathyroidism and 42.9 % in pseudohypoparathyroidism. No ectopic calcifications were observed through ultrasound. Calcium level varied from 1.13 to 2.37 mmol/L (1.61 ± 0.27 mmol/L). Serum PTH level of hypoparathyroidism varied from 1 to 59.18 pg/mL (12.28 ± 17.59 pg/mL); the level of pseudohypoparathyroidism varied from 99.39 to 868.00 pg/mL (436.10 ± 262.56 pg/mL). The alkaline phosphatase levels varied from 72 to 2094 IU/L. The EEG results were normal in 19 (45.24 %) patients and abnormal in 23 (54.76 %) patients; 29 (69.05 %) patients have subcortical calcification. Finally, Chvostek’s sign was positive in 22 (52.38 %) patients; likewise, Trousseau’s sign was positive in 13 (30.95 %) patients.

**Table 1:** Demographics and symptoms

	*n* = 42
Sex	
Male/female	22 (52.38 %)/20 (47.62 %)
Age of onset (years)	7.55 ± 5.21
Type of parathyroid disorders	
Primary	25 (59.52 %)
Pseudo	17 (40.48 %)
CT scan	
Subcortical zone calcification	29 (69.05 %)
Non-subcortical zone calcification	13 (30.95 %)
Chvostek’s sign	
Positive/negative	22 (52.38 %)/20 (47.62 %)
Trousseau’s sign	
Positive/negative	13 (30.95 %)/29 (69.05 %)
Results of EEG	
Normal/abnormal	19 (45.24 %)/23 (54.76 %)

Age of onset was shown as means ± SD; others were shown as *n* (%)

**Table 2:** Detailed description of seizures

Manifestations	Hypoparathyroidism	Pseudohypoparathyroidism
Mental retardation	6/25 (24 %)	2/17 (11.8 %)
Convulsions	18/25 (72 %)	10/17 (58.8 %)
Tetany	5/25 (20 %)	2/15 (11.8 %)
Numbness	1/25 (0.04 %)	0/17 (0 %)
Dizziness	0/25 (0 %)	1/17 (5.9 %)
Absence	0/25 (0 %)	1/17 (5.9 %)
AHO symptoms	0/25 (0 %)	4/17 (23.5 %)
QT interval prolongation	6/15 (40 %)	3/7 (42.9 %)

AHO: patients with this disorder are mainly manifested with short stature, characteristically shortened fourth and fifth metacarpals, rounded facies, and often mild mental retardation

### Effect of AEDs on seizures

The patients received different AEDs, such as levetiracetam (four patients), phenobarbital (two patients), carbamazepine (three patients), valproate (three patients), topiramate (two patients), and oxcarbazepine (two patients). Most of the patients underwent one-drug therapy; only two patients had their AEDs changed. One patient replaced carbamazepine with levetiracetam because of an allergy.

The results of univariate analysis are shown in Table 3. No significant differences were found in the variables between the AED and the non-AED treatment groups (sex, *P* = 0.82, OR = 1.64, 95 % CI = 0.31–4.40; serum calcium level, *P* = 0.76, OR = 1.99, 95 % CI = 0.03–158.80; results of EEG, *P* = 0.05, OR = 3.79, 95 % CI = 0.98–14.67; type of parathyroid disorders, *P* = 0.33, OR = 0.54, 95 % CI = 0.16–1.87; age of onset, *P* = 0.15, OR = 0.92, 95 % CI = 0.82–1.03; type of seizures, *P* = 0.62, OR = 0.69, 95 % CI = 0.16–3.05; results of CT scan, *P* = 0.66, OR = 1.34, 95 % CI = 0.37–4.93; Chvostek’s sign, *P* = 0.85, OR = 1.12, 95 % CI 0.35–3.57; and Trousseau’s sign, *P* = 0.81, OR = 1.18, 95 % CI = 0.29–4.76).

**Table 3:** Characteristics of 42 subjects divided into two groups according to use of AEDs

Factors	Treatment with AEDs	Treatment without AEDs	OR (95 % CI)	*P* value (two sided)
Sex			1.64 (0.31–4.40)	0.82
Male/female	7/7	15/13		
Serum calcium level (means ± SD)	1.59 ± 0.17	1.62 ± 0.32	1.99 (0.03–158.80)	0.76
Results of EEG			3.79 (0.98–14.67)	0.05
Normal/abnormal	3/11	16/12		
Type of parathyroid disorders			0.54 (0.16–1.87)	0.33
Hypoparathyroidism/pseudohypoparathyroidism	10/4	15/13		
Age of onset (means ± SD)	5.72 ± 6.03	8.47 ± 4.60	0.92 (0.82–1.03)	0.15
Type of seizures			0.69 (0.16–3.05)	0.62
Focal/generalized	6/4	12/12		
Results of CT scan			1.34 (0.37–4.93)	0.66
Subcortical calcification	9	20		
No subcortical calcification	5	8		
Chvostek’s sign			1.12 (0.35–3.57)	0.85
Positive/negative	7/7	15/13		
Trousseau’s sign			1.18 (0.29–4.76)	0.81
Positive/negative	4/10	9/19		

Conditional logistic regression was used for comparison

**Fig. 2:**
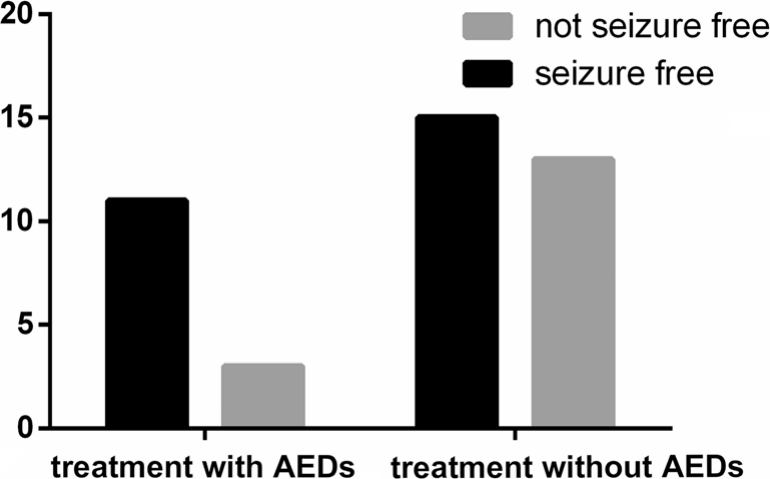
Seizure outcome is not significantly different between the subjects with AED treatment and those without AED treatment, *P* = 0.34

**Fig. 3:**
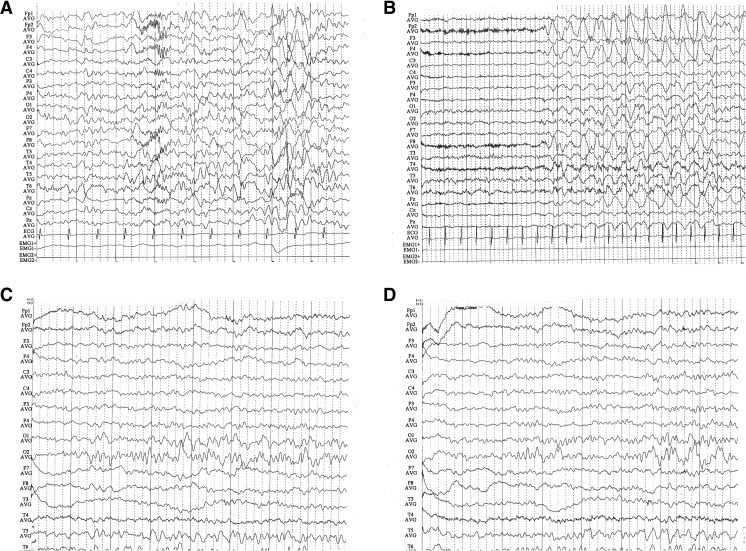
The EEG results of a patient with pseudohypoparathyroidism before (**a**, **b**) and after (**c**, **d**) calcium supplement therapy. Before treatment, the EEG shows suspicious epileptic discharge characterized by spike wave and multiple spike waves. After treatment, the epileptic discharge is obviously ameliorated and the patient has reached seizure-free state

As shown in Fig. 2 and Table 3, no significant differences were observed between the seizure outcomes after AED treatment and those of the controls for 1 year (*P* = 0.34, OR = 169.48, 95.0 % CI = 0.00–6,403,719.35). The seizure-free rates were 78.57 and 53.57 % in the AED and non-AED treatment groups, respectively. The therapeutic effect on a patient is shown in Fig. 3. The EEG result was significantly ameliorated after calcium supplement therapy for less than 1 week, and a seizure-free outcome was attained, and no recurrence was observed.

To confirm the necessity of AED use in patients with subcortical calcification, we subdivided the patients into AED and non-AED treatment groups before their seizure outcomes were compared as described previously, but with the patients matched at 1:1.

No significant differences were observed among all the observed variables (sex, *P* = 0.66, OR = 0.67, 95 % CI = 0.11–4.00; serum calcium level, *P* = 1, OR = 1, 95 % CI = 0.00–28,737.39; results of EEG, *P* = 0.22, OR = 4, 95 % CI = 0.45–35.79; type of parathyroid disorders, *P* = 0.42, OR = 0.5, 95 % CI = 0.09–2.73; age of onset, *P* = 0.47, OR = 0.95, 95 % CI = 0.81–1.10; type of seizures, *P* = 1, OR = 1, 95 % CI = 0.14–7.10; Chvostek’s sign, *P* = 1, OR = 1, 95 % CI = 0.20–4.96; Trousseau’s sign, *P* = 0.66, OR = 0.67, 95 % CI = 0.11–4.00).

**Fig. 4:**
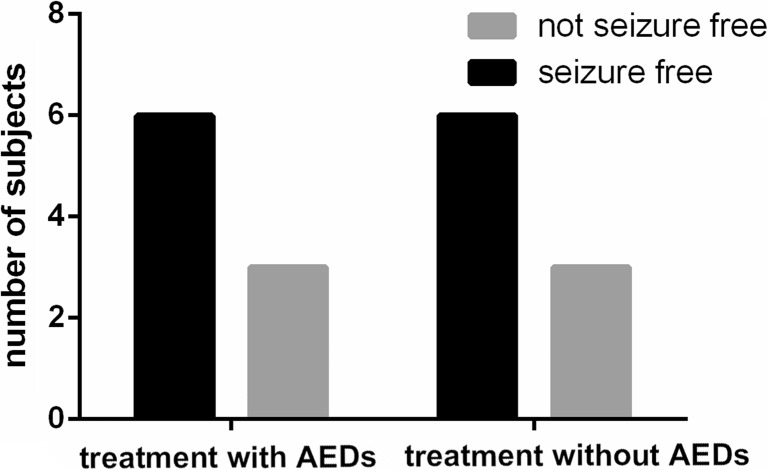
In the subjects with subcortical calcifications, seizure outcome is not significantly different in subjects with AED treatment and those without AED treatment, *P* = 0.38

In addition, the seizure outcomes were not different from the controls, as shown in Fig. 4 and Table 4 (*P* = 0.38, OR = 0.02, 95 % CI = 0.00–164.78). Seizure-free rates were 66.67 % in the AED and non-AED treatment groups.

**Table 4:** Characteristics of subjects with subcortical calcifications divided into two groups according to use of AEDs

Factor	Treatment with AEDs	Treatment without AEDs	OR (95 % CI)	*P* value (two sided)
Sex			0.67 (0.11–4.00)	0.66
Male/female	6/3	5/4		
Serum calcium level (means ± SD)	1.60 ± 0.22	1.79 ± 0.41	1 (0.00–28,737.39)	1
Results of EEG			4 (0.45–35.79)	0.22
Normal/abnormal	2/7	5/4		
Type of parathyroid disorders			0.5 (0.09–2.730	0.42
Hypoparathyroidism /pseudohypoparathyroidism	6/3	4/5		
Age of onset (means ± SD)	4.59 ± 5.49	6.78 ± 5.27	0.95 (0.81–1.10)	0.47
Type of seizures			1 (0.14–7.10)	1
Focal/generalized	3/3	4/5		
Chvostek’s sign			1 (0.20–4.96)	1
Positive/negative	4/5	4/5		
Trousseau’s sign			0.67 (0.11–4.00)	0.66
Positive/negative	3/6	2/7		

Conditional logistic regression was used for comparison

### Subcortical calcifications and EEG findings and seizures

The incidence of frontal lobe calcification is the highest among all subcortical calcifications. In our study, only 6 patients had frontal lobe calcifications, 19 patients had subcortical calcifications in their frontal lobes and other sites, and 3 patients had no frontal lobe calcification. These findings indicated that the incidence of frontal lobe calcification is 89.3 %.

**Fig. 5:**
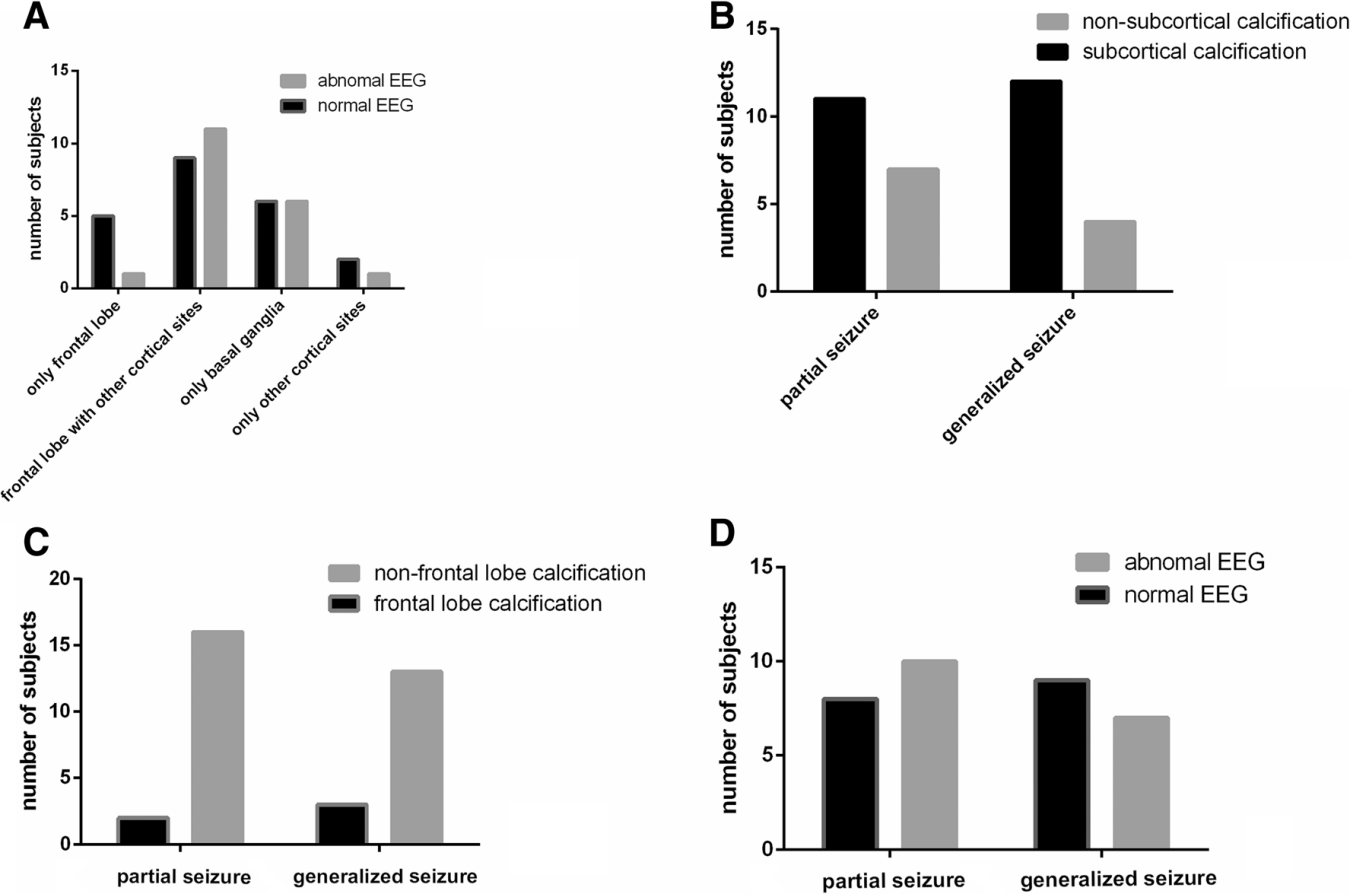
Relationship among results of CT scan and EEG findings and type of seizures. No significant association was observed (**a** sites of calcifications × EEG, *P* value not available; **b** with or without subcortical calcifications × type of seizures, *P* = 0.477; **c** with or without frontal lobe calcifications × type of seizures, *P* = 0.648; **d** EEG findings × type of seizures, *P* = 0.732)

In addition, the relationship between the EEG and the CT scan results is shown below: the ratio of the abnormal/normal EEG findings is 50 % in the case with only basal ganglia calcifications. This result is consistent with the conclusion that abnormal EEG findings are not associated with basal ganglia calcifications [8]. The ratio is reduced to 20 % in the case with only frontal lobe calcifications, and 50 % ratio was observed in the case with subcortical calcifications except in the frontal lobe. The ratio is 55 % in cases with subcortical calcifications in the frontal lobe and other sites. The result is shown in Fig. 5.

In the results of the chi-square test, the type of seizure was not significantly associated with either the subcortical calcifications or the EEG findings (subcortical calcifications and type of seizures, *P* = 0.477; EEG findings and type of seizures, *P* = 0.732). In addition, the type of seizures did not change significantly regardless of the existence of frontal lobe calcifications (Fig. 5).

### Calcium level and seizure outcome

**Fig. 6:**
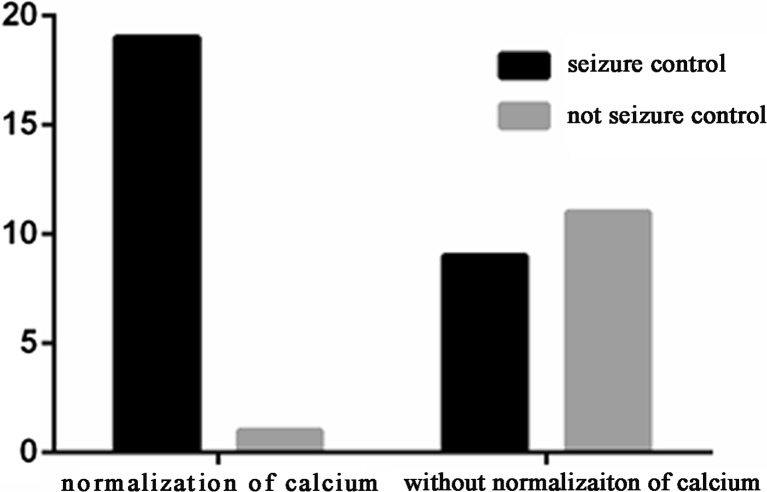
Seizure control outcomes in untreated patients with or without normalization of calcium

The outcomes of seizure control were evaluated in all the untreated patients according to calcium normalization. A matched case–control comparison was performed by sex and duration of disease. The seizure control outcome was evaluated when calcium normalization occurred. The results suggested that the rate of seizure control was relatively high in patients with calcium normalization (*P* = 0.006; Fig. 6).

### Intracranial calcifications and seizure features and outcomes

Among the 120 patients, we selected the patients with calcifications at different sites to compare their seizure features and seizure control outcomes.

As shown in Table 5, no significant difference can be observed among the seizure durations (calcification, 3.86 ± 5.50 with *n* = 43 vs. no calcification, 10.72 ± 28.68 with *n* = 17; *P* = 0.341). However, the seizure frequency was relatively high (calcification, 597.55 ± 964.08 with *n* = 22 vs. no calcification, 1654.00 ± 1290.34 with *n* = 11; *P* = 0.013). The duration since first seizure was relatively short (calcification, 5.67 ± 8.18 with *n* = 73 vs. no calcification, 1.17 ± 2.29 with *n* = 31; *P* = 0.00) in the no-calcification group.

**Table 5:** Comparison of seizure features between patients with or without calcifications

	Calcifications		*P* value
	Yes	No	
Seizure duration (min)	3.86 ± 5.50 (*n* = 43)	10.72 ± 28.68 (*n* = 17)	0.341
Frequency (/year)	597.55 ± 964.08 (*n* = 22)	1654.00 ± 1290.34 (*n* = 11)	0.013
Duration since first seizure (years)	5.67 ± 8.18 (*n* = 73)	1.17 ± 2.29 (*n* = 31)	0.00

As shown in Table 6, no significant difference was present between the multiple calcification group and the single calcification group with respect to seizure duration (multiple calcifications, 2.70 ± 2.92 with *n* = 29 vs. single calcification, 6.24 ± 8.38 with *n* = 14; *P* = 0.147), seizure frequency (multiple calcifications, 643.87 ± 1049.21 with *n* = 13 vs. single calcification, 530.64 ± 883.21 with *n* = 9; *P* = 0.794), and duration since the first seizure (multiple calcifications, 5.66 ± 8.59 with *n* = 55 vs. single calcification, 5.68 ± 7.01 with *n* = 18; *P* = 0.995).

**Table 6:** Comparison of seizure features between patients with and without multiple calcifications

	Multiple calcifications		*P* value
	Yes	No	
Seizure duration (min)	2.70 ± 2.92 (*n* = 29)	6.24 ± 8.38 (*n* = 14)	0.147
Frequency (/year)	643.87 ± 1049.21 (*n* = 13)	530.64 ± 883.21 (*n* = 9)	0.794
Duration since first seizure (years)	5.66 ± 8.59 (*n* = 55)	5.68 ± 7.01 (*n* = 18)	0.995

As shown in Table 7, there was no significant difference with respect to seizure duration (only basal ganglion calcification, 6.03 ± 9.03 vs. no calcification, 10.72 ± 28.64; *P* = 0.59) and frequency (only basal ganglion calcification, 528.59 ± 944.17 vs. no calcification, 1654.00 ± 1290.34; *P* = 0.052) between the basal ganglion calcification group (*n* = 16) and the no-calcification group (*n* = 31). Duration since first seizure was relatively short in the no-calcification group (only basal ganglion calcification 4.95 ± 6.40 vs. no calcification, 1.17 ± 2.29; *P* = 0.035).

**Table 7:** Comparison of seizure features between patients with basal ganglion calcification and patients without calcification

	Only basal ganglion calcification (*n* = 16)	Without calcification (*n* = 31)	*P* value
Seizure duration (min)	6.03 ± 9.03	10.72 ± 28.64	0.59
Frequency (/year)	528.59 ± 944.17	1654.00 ± 1290.34	0.052
Duration since first seizure (years)	4.95 ± 6.40	1.17 ± 2.29	0.035

## Discussion

Clinical seizures are present in about two thirds of idiopathic hypoparathyroidism incidence. Their symptoms vary from circumoral numbness, muscle cramps, paresthesias of the hands and feet, to generalized tonic–clonic seizures. Seizures can be induced by hypocalcemia or intracranial calcifications, but whether these seizures are epilepsy or tetany cannot be determined from the clinical manifestations alone. To clarify the type of seizures, we must evaluate the EEG to determine whether epileptic discharges are present; calcium supplement therapy should also confirm whether seizures respond to this treatment [9].

According to our results, as the first symptom of hypoparathyroidism in children, epileptic seizures were not evidently improved by administering AED treatment to calcium supplement therapy.

Even in the presence of cortical calcifications, which could be suspected as a cause of structural epilepsy, AEDs did not significantly improve the seizure outcome. This finding can be explained as follows: the manifestations of parathyroid disorders were mainly caused by calcium phosphorus metabolic disorders, and the intracranial calcifications were metastatic calcifications (calcifications that occurred due to hypocalcemia but not due to intracranial lesion). Treatment of these diseases must focus on calcium supplementation and not on the invalid AED therapy. Our results are consistent with the study of Modi et al. [10], who indicated that patients with hypoparathyroidism could reach optimal seizure outcomes regardless of the use of AEDs. Although adult patients were included in their study, patients with seizures as the first manifestation were mainly in their childhood; this trend was similar among our included patients.

Serum calcium levels are low in patients with or without AED treatment so these factors could trigger seizures. The baselines of the two groups are not significantly different from each other, including the serum calcium. Therefore, the statement that the seizure control outcome does not have statistical or clinical significance is reasonable.

Among the varied follow-up durations of all the patients in this study, we chose the 1-year follow-up because the start of the AED treatment was used as reference to evaluate the efficacy of AEDs on seizure control. Runge [11] evaluated the effectiveness of lacosamide for almost 6 months since its addition to therapy. Hussain [12] evaluated the effectiveness of cannabidiol (CBD) as an add-on therapy for patients with infantile spasms (IS) or Lennox–Gastaut (G) syndrome at ∼6.8 months after medication started. Chen [13] evaluated the efficacy of levetiracetam in patients with electrical status epilepticus in sleep (ESES); the follow-up duration was used as inclusion criteria because his study started no less than 1 year since patient medication. Thus, 1-year-long follow-up is enough to determine the effectiveness of AED on seizure control in this study.

Cortical calcifications mainly occur in the frontal lobe. Moreover, EEG findings are not associated with the type of seizures, thereby supporting our conclusion previously. The rate of abnormal EEG findings is relatively low when only the subcortical calcifications are present in the frontal lobe or the other sites because of the small sample size and the false-negative rate of EEG. As referenced previously, discrepancies exist on the relation between the subcortical calcification and the abnormal EEG findings, and numerous studies are limited to a certain disease (for example, brain cysticercosis and Sturge–Weber syndrome) [5–7, 14]. Our sample size is small, and this study is just a preliminary exploration of this problem in parathyroid disorders.

Serum calcium levels are lower in patients with AED treatment. In addition, most patients were taking one of the following AEDs: phenobarbital, carbamazepine, or valproic acid sodium; these AEDs are detrimental to calcium levels and patient prognosis [15]. These results suggest that the use of AEDs must be carefully considered in patients with parathyroid disorders because these drugs could be ineffective or even detrimental.

Given that some of the detailed EEG results cannot be obtained in our patients, the analysis of a detailed EEG description is not listed previously. However, according to our data, the description of EEG is consistent with previous publications, thereby indicating that the abnormalities in the EEG are characterized by slow theta and delta waves, spike-wave discharges that vary in frequency from 1.5 c/s to 4 c/s, and single and multiple spiking [16].

By excluding the effect of AED therapy, calcium normalization facilitated seizure control in all the patients without AED treatment. This result confirmed the nature of hypoparathyroidism as a metabolic disorder and supports the finding that AED treatment cannot improve the seizure control outcome.

**Fig. 7:**
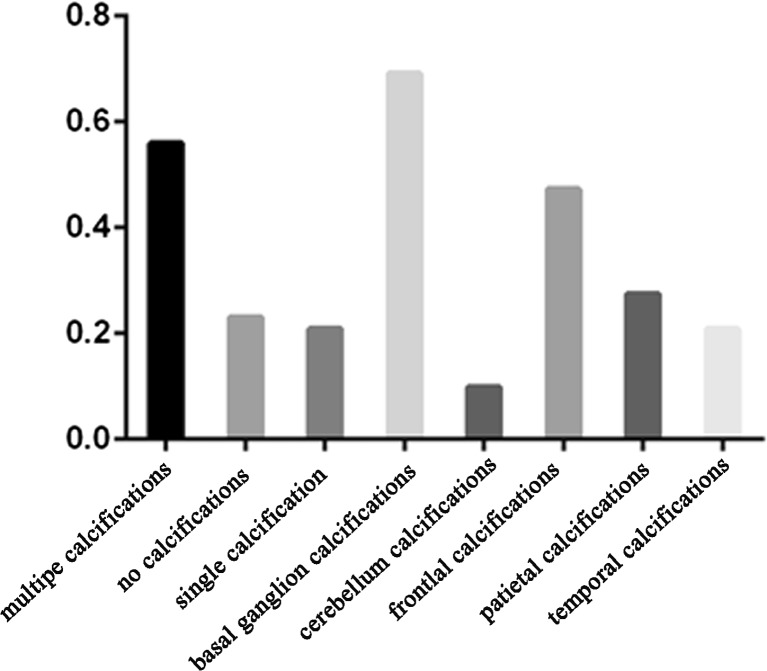
Intracranial calcifications in patients with poorly controlled calcium

As reported in a previous study [17], 21.5 to 73 % of the patients showed intracranial calcifications. The intracranial calcification rate of 76.9 % in patients with poorly controlled calcium levels must be extremely high (Fig. 7), thereby indicating that calcification is associated with long-term poorly controlled calcium. Almost half of the patients with poorly controlled calcium levels did not achieve seizure control. This trend is consistent with the rate of seizure control in patients without AED treatment and calcium normalization (Fig. 6).

Patients with calcification showed relatively long seizure durations since the first seizure. As shown in Tables 5 and 7, the duration since the first seizure is relatively long in patients with calcifications. In Table 6, both groups have intracranial calcifications and the duration was not significantly different since the first seizure was observed. This finding is consistent with the statement that calcification is a long-term complication in disorders of calcium phosphorus metabolism [17].

Basal ganglion calcification is not associated with epileptic discharges [8]. No significant differences were observed when the seizure features were compared between patients with only basal ganglion calcification and patients without calcification, except in the duration since the first seizure.

Consistent conclusions cannot be formulated by comparing the seizure frequencies between calcification and no-calcification groups, as well as between multiple calcification and single calcification groups. In the multiple calcification group and calcification group, calcification in the different sites probably had varying effects on epileptic discharges. Except for these confounding factors, the sample size of these comparisons is small. Thus, further investigation is required for more conclusive results.

This work is a retrospective study with a small sample size. Therefore, selection bias may exist. Large-sample prospective studies are necessary to elucidate the necessity of administering AEDs in parathyroid disorders.
